# Anakinra versus Baricitinib: Different Strategies for Patients Hospitalized with COVID-19 †

**DOI:** 10.3390/jcm10174019

**Published:** 2021-09-06

**Authors:** José A García-García, Marta Pérez-Quintana, Consuelo Ramos-Giráldez, Isabel Cebrián-González, María L Martín-Ponce, José del Valle-Villagrán, María A Navarro-Puerto, Jorge Sánchez-Villegas, Rocío Gómez-Herreros, Isabel Manoja-Bustos, Daniel León-Martí, Lucía Serrano-Rodríguez, Alejandra de Miguel-Albarreal, María J Velasco-Romero, Francisco Mula-Falcón, Pilar Fernández-Pérez, Isabel Melguizo-Moya, María J Pérez-Quintana, Guillermo Romero-Molina, Salvador Vergara-López, José L Marenco-de la Fuente, Jorge Marín-Martín, José A Mira-Escarti

**Affiliations:** 1 Department of Internal Medicine, Valme University Hospital, 41014 Seville, Spain; mlmartinponce@gmail.com (M.L.M.-P.); masunnp@hotmail.com (M.A.N.-P.); rotxogh@yahoo.es (R.G.-H.); dani17893@gmail.com (D.L.-M.); lexis11s@gmail.com (A.d.M.-A.); framulfal@gmail.com (F.M.-F.); imelguizo@yahoo.es (I.M.-M.); guirommol@hotmail.com (G.R.-M.); vergara_lopez@hotmail.com (S.V.-L.); miraescarti@yahoo.es (J.A.M.-E.); 2 Department of Internal Medicine, Osuna Hospital, 41640 Seville, Spain; pquintana.marta@gmail.com (M.P.-Q.); iscego1@gmail.com (I.C.-G.); jdelvalle79@gmail.com (J.d.V.-V.); jsvilleg@gmail.com (J.S.-V.); isa_91_mlg@hotmail.com (I.M.-B.); luciaserranorodriguez@gmail.com (L.S.-R.); mariajose_vr@live.com (M.J.V.-R.); pilagould91@gmail.com (P.F.-P.); jorge.marin.sspa@juntadeandalucia.es (J.M.-M.); 3 Department of Rheumatology, Valme University Hospital, 41014 Seville, Spain; cramosgiraldez@gmail.com (C.R.-G.); mariajoseperezquintana@gmail.com (M.J.P.-Q.); jmarenco@gmail.com (J.L.M.-d.l.F.)

**Keywords:** COVID-19, anakinra, baricitinib, corticosteroids, mortality

## Abstract

Background: Immunomodulatory drugs have been used in patients with severe COVID-19. The objective of this study was to evaluate the effects of two different strategies, based either on an interleukin-1 inhibitor, anakinra, or on a JAK inhibitor, such as baricitinib, on the survival of patients hospitalized with COVID-19 pneumonia. Methods: Individuals admitted to two hospitals because of COVID-19 were included if they fulfilled the clinical, radiological, and laboratory criteria for moderate-to-severe disease. Patients were classified according to the first immunomodulatory drug prescribed: anakinra or baricitinib. All subjects were concomitantly treated with corticosteroids, in addition to standard care. The main outcomes were the need for invasive mechanical ventilation (IMV) and in-hospital death. Statistical analysis included propensity score matching and Cox regression model. Results: The study subjects included 125 and 217 individuals in the anakinra and baricitinib groups, respectively. IMV was required in 13 (10.4%) and 10 (4.6%) patients, respectively (*p* = 0.039). During this period, 22 (17.6%) and 36 (16.6%) individuals died in both groups (*p* = 0.811). Older age, low functional status, high comorbidity, need for IMV, elevated lactate dehydrogenase, and use of a high flow of oxygen at initially were found to be associated with worse clinical outcomes. No differences according to the immunomodulatory therapy used were observed. For most of the deceased individuals, early interruption of anakinra or baricitinib had occurred at the time of their admission to the intensive care unit. Conclusions: Similar mortality is observed in patients treated with anakinra or baricitinib plus corticosteroids.

## 1. Introduction

Two processes may occur in SARS-CoV-2 infection that causes COVID-19. First, viral replication predominates. Subsequently, a subset of patients may develop hyperinflammation, leading to moderate or severe COVID-19 pneumonia [1,2]. A cytokine storm is caused by excessive immune reactions and has been recognized as a pathophysiologic mechanism in severe COVID-19 [3]. Therefore, blocking the hyperimmune response and the secondary cytokine storm is critical for the treatment of severe COVID-19. Corticosteroids have been used to control the hyperimmune state. In fact, dexamethasone, at a dose of 6 mg once daily, has been shown to reduce the mortality of patients with severe COVID-19 pneumonia [4]. Moreover, pulses of methylprednisolone have also been demonstrated to be effective in treating cases of COVID-19 characterized by a strong inflammatory profile and severe respiratory symptoms [5,6]. 

Several immunomodulatory drugs have also been considered in the treatment of COVID-19, including recombinant human interleukin (IL) inhibitors [7], such as tocilizumab and anakinra, or the Janus kinase (JAK) inhibitor baricitinib [8]. An agent blocking the IL-6 receptor, tocilizumab, was one of the first immunomodulatory therapies to be proposed, given the fact that higher IL-6 concentrations have been associated with worse outcomes in patients with COVID-19 [9,10,11]. However, different studies with tocilizumab, including clinical trials, have revealed mixed results [12,13,14,15,16]. A central role of IL-1 in the inflammatory response has also been described [17]. In this sense, anakinra, a recombinant IL-1-receptor antagonist (IL-1ra), has been proposed as a potential therapeutic in severe COVID-19. It is well tolerated, has only mild immunosuppressive effects, and can be easily administered subcutaneously [18]. In addition, anakinra decreases IL-6 production because IL-1 is a potent inducer of IL-6 [17]. Therefore, the suggested beneficial effects of tocilizumab are also expected to be observed with anakinra. The published data for anakinra are based on a few observational studies with different designs, regimens, and concomitant or non-concomitant corticosteroid therapy [19,20,21,22,23]. However, further validation through ongoing randomized clinical trials is needed. In the hyperinflammatory syndrome, JAK–signal transducer and activator of transcription (STAT) signaling plays an important role in the pro-inflammatory cytokine-mediated signaling process [24]. Baricitinib is a potent and selective JAK inhibitor, requiring once-daily oral dosing and having an acceptable side-effect profile [25]. Baricitinib has a double effect against severe COVID-19. It inhibits the entry of SARS-CoV-2 into the target cells and blocks the induction of cytokine storms by suppressing JAK1/JAK2 [25]. In recent observational studies, baricitinib was associated with greater improvement in pulmonary function [26] and a reduction in mortality rate and intensive care unit (ICU) admissions in patients with moderate-to-severe COVID-19 [27]. In a randomized trial, the use of baricitinib plus remdesivir was superior to remdesivir alone in reducing recovery time and accelerating improvements in clinical status among patients with COVID-19 [28]. However, no consistent data have published about the relationship of baricitinib with hard clinical outcomes to date. Based on the above pathophysiological hypothesis, baricitinib could be used early in COVID-19 patients to inhibit SARS-CoV-2 entry into target cells. However, anakinra does not appear to be effective in non-severe infections. In fact, a randomized controlled trial was stopped early because anakinra did not improve outcomes in patients with mild COVID-19 pneumonia [29].

The aim of this study was to compare the efficacy in terms of the need for IMV and the mortality of two different strategies, based either on anakinra or on baricitinib therapies, applied to patients hospitalized with COVID-19 pneumonia.

## 2. Materials and Methods

### 2.1. Design and Patients

Our retrospective cohort included all patients who were admitted to internal medicine units in two tertiary healthcare centers in Seville (southern Spain) because of moderate-to-severe COVID-19 from the beginning of September to the end of November 2020. The inclusion criteria were as follows: (i) age over 18 years with SARS-CoV-2 infection indicated by PCR or the presence of antigen in nasopharyngeal swab; (ii) the use of immunomodulatory drugs; (iii) one of the following criteria suggestive of lower respiratory tract infection at the time of enrolment—lung infiltrates on a chest X-ray and/or computed tomography scan or hypoxemia, defined as requiring any oxygen (O_2_) support to achieve O_2_ saturation of >93%; and (iv) at least one of the following laboratory criteria—C-reactive protein (CRP) > 50 mg/L, ferritin > 500 ng/mL, D-dimer > 500 ng/mL, or lactate dehydrogenase (LDH) > 250 U/L.

Clinical data were recorded daily from all consecutive patients admitted to the hospital for COVID-19 in their electronic records. The same physicians collected this information from patients’ records, and they were manually entered by clinicians in a specific database.

**Figure 1:**
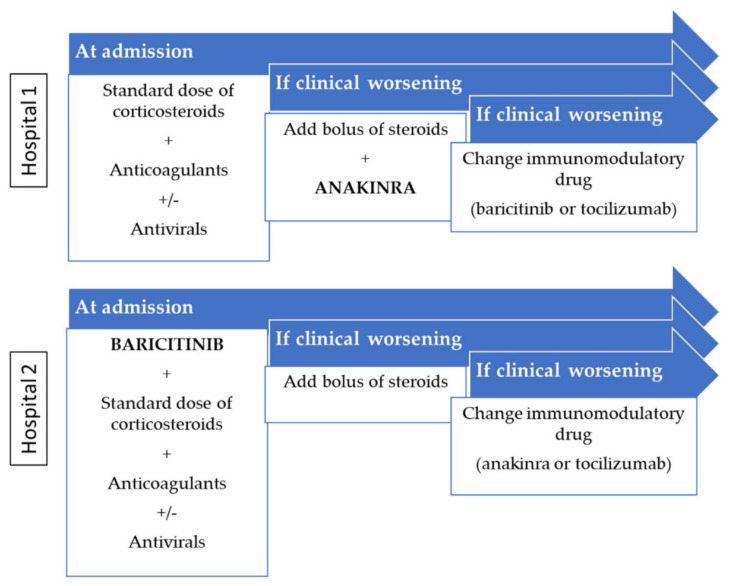
Internal medicine protocols in hospitalized patients with COVID-19.

A different strategy was implemented in each hospital according to its units’ protocols based on anakinra or baricitinib as the first immunomodulatory drug recommended in that hospital. Both protocols included corticosteroid and anticoagulant therapies (Figure 1). Thus, patients were classified in each arm according to the first immunomodulatory drug used.

Patients were excluded if a major clinical event (IMV or death) or a change in the immunomodulatory drug occurred before two consecutive doses of baricitinib (48 h) or anakinra (24 h) had been administered.

### 2.2. Variables and Follow-Up

The primary endpoint was in-hospital mortality, assessed by time-to-event analysis. The secondary outcomes were the need for invasive mechanical ventilation and the change in category based on the ordinal score of a modified WHO progression scale from baseline to the censored data according to supplemental oxygen.

All patients were censored at discharge from the hospital or on the date of death if this occurred first. Therefore, data included hospitalization in conventional and intensive units. Demographic information (sex, age, residence), baseline comorbidities measured by the Charlson index and a performance measure of activities of daily living by the Barthel scale, laboratory tests at the beginning of immunomodulatory therapy (CRP, ferritin, D-dimer, LDH, liver aminotransferases, platelet and lymphocyte counts), and COVID-19 treatment (antivirals, corticosteroids, immunomodulatory and anticoagulant drugs) were collected. Details regarding the need to change to another immunomodulatory drug, if applicable, and the cause of this were also included.

The World Health Organization working group on the clinical characterization and management of COVID-19 developed a minimum set of common outcome measures for studies of COVID-19. This set included a measure of clinical progression based on the WHO clinical progression scale [30]. However, hospitalized patients requiring supplemental oxygen without intubation are not classified properly, because categories 5 and 6 include a wide range of non-ICU individuals, ranging from those with mild disease requiring low-flow oxygen to severe cases with non-invasive ventilation. Thus, we recategorized our patients into specific subsets (Table 1). Based on this classification, the oxygen therapy requirements at the initiation of immunomodulatory therapy and the maximum supplemental oxygen used during follow-up were also recorded.

Comorbidities were calculated using the Charlson index [31]. The Barthel scale was used to measure performance for 10 items about activities of daily living [32]. To interpret the Barthel scale values, they were categorized into 5 groups: total dependency (0–20 points), severe (21–35), moderate (40–55), slight (60–85), and no dependency (90–100). The laboratory tests included determination of lymphocyte and platelet counts; LDH, serum ferritin, alanine aminotransferase (ALT), CRP, and D-dimer levels; and the erythrocyte sedimentation rate.

### 2.3. Treatments

Anakinra was administrated subcutaneously at a standard dose of 200 mg twice on the first day, followed by 100 mg twice daily until a course of 10 days had been completed. The dose of this drug was adjusted to half if the renal glomerular filtration rate was under 30 mL/min/1.73 m^2^.

Baricitinib was administrated orally at a standard dose of 4 mg once a day for up to 10 days. In the same way, the dose was adjusted to half if the renal filtration rate was under 60 mL/min/1.73 m^2^.

Other therapies were administered to both groups as concomitant treatments based on the physician’s criteria, including antiviral drugs, corticosteroids, and anticoagulant therapy to prevent coagulopathic complications. Methylprednisolone or dexamethasone at a once-daily dose equal to or higher than 125 or 20 mg was considered as pulses of steroids and was administered for 3 or more days.

### 2.4. Statistical Analysis

Continuous variables were presented as medians and interquartile ranges (IQRs) and categorical variables as absolute (*n*) or relative (%) frequencies. We applied the chi^2^ test and Student’s *t*-test (or the Mann–Whitney test if the variables had non-normal distributions) to assess the differences in the clinical outcomes according to the type of variables.

Propensity score matching was used to adjust for some baseline characteristics with differences between them. A standardized difference of <0.2 as the upper limit of acceptable imbalance in baseline covariates was calculated.

We calculated the rates of intubation and death in the anakinra and control groups by a time-to-event analysis. The association of the main variables with time-related endpoints was analyzed using Kaplan–Meier curves and Cox regression analysis. Statistically significant differences were considered when *p* < 0.05. However, any variable with *p* < 0.1 in the univariate model was included in multivariate analysis.

Statistical analyses were performed using IBM SPSS software version 25 (SPSS, Chicago, IL, USA).

## 3. Results

**Figure 2:**
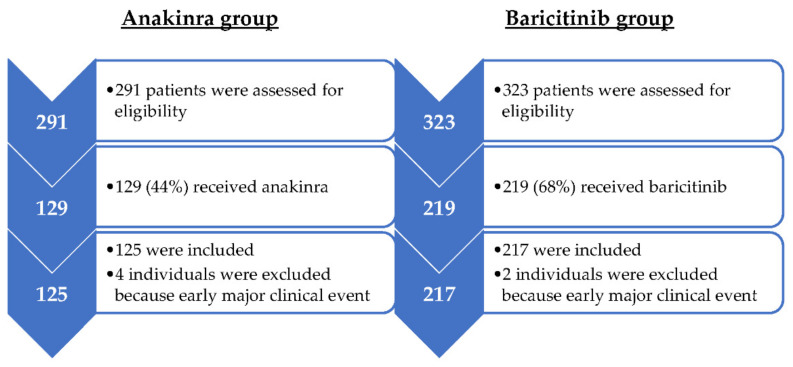
Flowchart of patients included.

During the period of the study, 291 and 323 patients were admitted in the internal medicine ward of two different hospitals. Of them, 129 (44.3%) and 219 (67.8%) patients were treated with anakinra and baricitinib, respectively. Six patients were excluded because early major clinical events occurred: four in the anakinra group due to intubation before 24 h at the initiation of anakinra and two subjects in the baricitinib group because of death before the first 48 h under treatment (Figure 2).

Propensity scores were calculated based on the patients’ following baseline characteristics: comorbidity index, Barthel scale, high-flow oxygen on presentation, dexamethasone at admission, and baseline CRP. After matching, there was a total of 93 subjects within each group.

### 3.1. Baseline Characteristics

The mean age of the total cohort was 69.4 years, and 57.6% were male. Total, severe, or moderate grade of dependency (Barthel scale < 60 points) was observed in 12.3% of patients, while more than two comorbidities were present in 54.7% of patients. Demographic, laboratory, and clinical data of both groups are shown in Table 2. A higher rate of individuals with comorbidities and dependency in the baricitinib group was observed. By contrast, the subjects in the anakinra group showed a significant elevation in several biomarkers of inflammation at the beginning of therapy, such as CRP, ALT, and LDH (Table 2). Statistically relevant values are highlighted in bold in Table 2.

### 3.2. Treatments

Antiviral therapy was less common in the anakinra arm than in the baricitinib arm, including remdesivir or lopinavir/ritonavir (2% vs. 51%, *p* < 0.001), respectively. All the patients were treated with corticosteroids in both groups. However, dexamethasone at admission was used more frequently among individuals under baricitinib treatment (62% vs. 79%, *p* < 0.001) as the first corticosteroid used. By contrast, all the patients in the anakinra group received high doses of corticosteroids as a concomitant therapy if clinical worsening was observed.

Immunomodulatory drugs were switched in 5 (4%) and 31 (14.3%) subjects in the anakinra and baricitinib groups, respectively, because they were considered non-effective. In the anakinra group, baricitinib was used in four individuals and tocilizumab in the remaining individuals. In the baricitinib group, anakinra and tocilizumab were prescribed for 18 and 13 subjects when clinical conditions worsened, respectively. Time from the start to switch the first immunomodulatory drug was 8 (5–9) days and 5 (2–7) days in anakinra and baricitinib groups, respectively (*p* < 0.001). More details about the treatment used are shown in Table 2.

**Figure 3:**
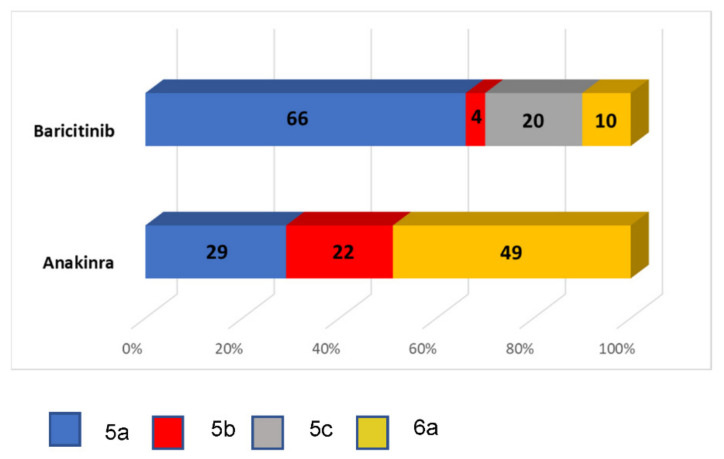
Bar plots at the beginning of immunomodulatory drug treatment according to the modified WHO clinical progression scale.

Patients in the anakinra arm needed higher levels of oxygen support at day 0 than those in the baricitinib group (Figure 3). Supplemental oxygen with high-flow oxygen, ≥5 lpm (category 5b or more), was required at baseline in 70.5% and 34.4% of the individuals in the anakinra and baricitinib groups, respectively. However, among these patients with severe infection, 36.3% and 45.3% (difference 9.0%, *p* < 0.001) of the subjects worsened by one or more steps during hospitalization based on the modified ordinal scale.

### 3.3. Outcome Events

In the anakinra and baricitinib original groups without matching, 13 (10.4%) and 10 (4.6%) patients required IMV, respectively (*p* = 0.039). Meanwhile, 22 (17.6%) and 36 (16.6%) patients died during this period, respectively (*p* = 0.811). When both events were analyzed together, 25 (20%) vs. 39 (18%) subjects needed IMV or died during follow-up (*p* = 0.643). According to the propensity score, 7 (7.5%) and 7 (7.5%) patients required IMV, respectively (*p* = 1). In terms of mortality, 15 (16.1%) and 21 (22.6%) patients died during this period, respectively (*p* = 0.811).

**Figure 4:**
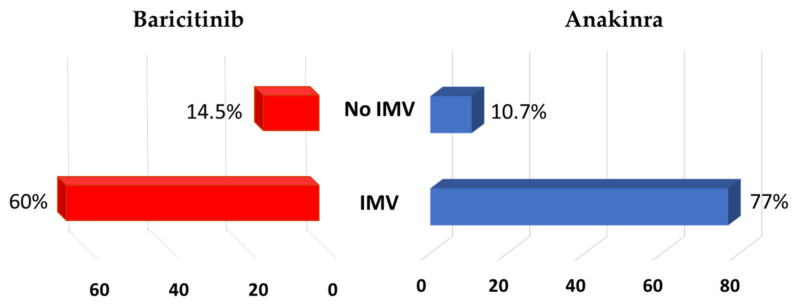
Mortality according to requiring invasive mechanical ventilation (IMV).

Depending on the need for intubation, 10 (77%) of 13 and 6 (60%) of 10 individuals who required IMV died in the anakinra and baricitinib groups, respectively (*p* = 0.382). By contrast, among the subjects who did not need intubation, 12 (10.7%) of 112 and 30 (14.5%) of 207 died, respectively (*p* = 0.341) (Figure 4). The median times receiving immunomodulatory drugs before needing intubation were 3 (2–6) and 4 (3–7) days among the anakinra and baricitinib patients, respectively. All eight individuals died when anakinra was discontinued in the first 3 days after intubation. However, three (60%) of five subjects in the ICU with more than 3 days of anakinra treatment survived. By contrast, five (83%) of six intubated and deceased individuals in the baricitinib group were treated for less than 3 days with this drug in the ICU.

Mortality was higher in patients who were in higher categories according to the ordinal modified scale when treatment with the immunomodulatory drug began. The mortality of the patients classified at the beginning in category 5a or 5b (FiO_2_ < 35%) vs. category 5c or more (FiO_2_ ≥ 35%) was 6.2% vs. 29.5% (*p* < 0.001) in the anakinra group and 13.3% vs. 23.9% (*p* = 0.048) in the baricitinib group, respectively.

#### 3.3.1. Immunomodulatory Drug

**Figure 5:**
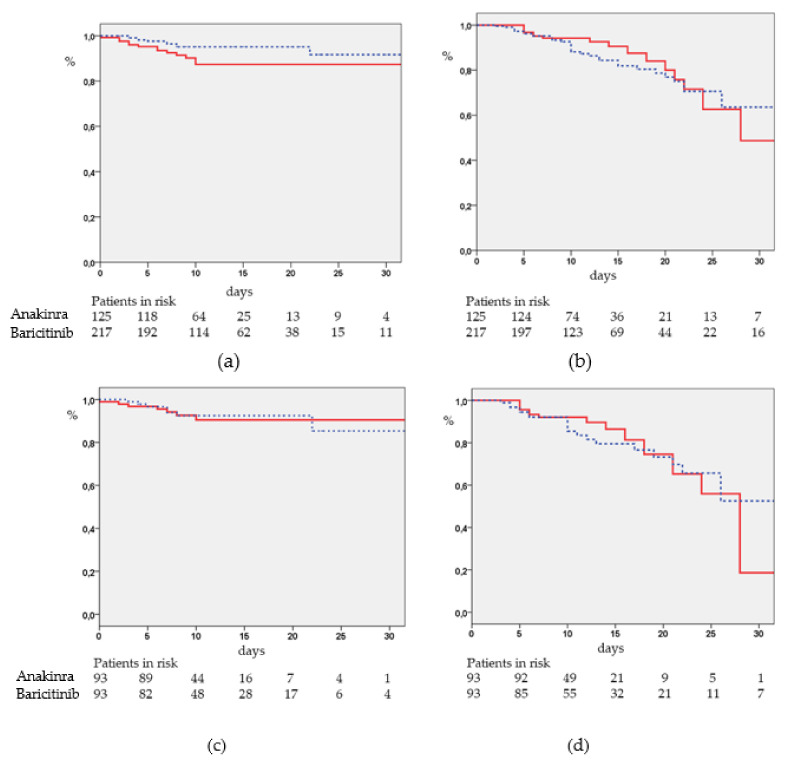
Probability of remaining free of invasive mechanical ventilation (**a**) and death (**b**) in the anakinra (continuous line) and baricitinib (dashed line) groups. Kaplan–Meier curves for IMV (**c**) and mortality (**d**) according to the immunomodulatory drugs and matching populations.

The Kaplan–Meier curves for the primary endpoints according to the immunomodulatory drugs are shown in Figure 5. The estimated median intubation-free periods (95% confidence interval (CI)) (Figure 5a) were 66.3 (61.7–70.8) and 83.5 (78.4–88.7) days in the anakinra and baricitinib groups, respectively (*p* = 0.044). The median survival periods (95% CI) were 42.3 (30.1–54.5) vs. 74.5 (51.7–97.3) days (*p* = 0.675) in the anakinra and baricitinib groups, respectively (Figure 5b). Among matching individuals, no differences were found in the frequency of IMV or deaths (Figure 5c,d).

Based on changes in the immunomodulatory drug when it was considered non-effective, only one (25%) of the four patients who switched from anakinra to baricitinib survived. The only subject in whom anakinra was changed to tocilizumab also died. In the baricitinib group, 12 (66%) and 7 (54%) individuals survived after switched from baricitinib to anakinra or tocilizumab, respectively.

#### 3.3.2. Multivariate Analysis

High levels of LDH (*p* = 0.027) and the need for oxygen supplementation with masks with reservoir bags at the beginning of immunomodulatory drug treatment (*p* = 0.036) were associated with intubation. Dexamethasone at baseline is a protective factor in intubation (Table 3).

By contrast, the need for intubation (*p* < 0.001), age older than 70 years (*p* < 0.001), dependency as indicated by a Barthel index value less than 60 (*p* = 0.037), and the use of pulses of corticosteroids (*p* < 0.001) were associated with a higher proportion of mortality (Table 4). Survival was not related to the use of dexamethasone at baseline (*p* = 0.532). No clinical events were related to the choice of the first immunomodulatory drug.

The multivariate analysis for 186 matching patients showed similar results. The Barthel score, Charlson index, IMV, older age, and high-flow oxygen at admission were associated with mortality (Table 5). Survival was not related to the use of pulses of corticosteroids.

#### 3.3.3. Adverse Events

In terms of related symptomatic adverse events, bowel perforation was observed in a patient treated in the anakinra group, but invasive diagnostic and therapeutic procedures were rejected because of the basal functional status. In terms of infections, 15 (12%) and 36 (16.6%) cases of bacterial pneumonia infection were suspected during the hospital stays in the anakinra and baricitinib groups, respectively (*p* = 0.351). Bacteriemia was diagnosed in six (4.8%) and seven (3.2%) subjects, respectively. Delirium was observed more frequently in the anakinra group compared to the baricitinib group(15 (12%) vs. 10 (4.6%), *p* = 0.011). Finally, 5 (4%) and 11 (5.1%) individuals in the anakinra and baricitinib groups developed heart complications, respectively (4 and 10 new arrhythmias, respectively, and one myocardial infarction in each group).

## 4. Discussion

This was a retrospective observational study investigating two different therapeutic strategies based on two immunomodulatory drugs: anakinra and baricitinib. Our findings can be summarized as follows: (i) Similar mortality was observed in both populations, and (ii) older age, high-flow oxygen at baseline, low functional status, high comorbidity, and the need for IMV were found to be associated with reduced survival.

To date, the experience with anakinra in patients with COVID-19 is limited and it is based on mainly small observational studies [19,20,21,22,23]. To the best of our knowledge, this study reports on the largest number of patients with COVID-19 treated with anakinra to date. Mixed results have previously been reported. Admission to the ICU for invasive mechanical ventilation support occurred for more than 27% of patients treated with anakinra in three previous studies [19,21,22]. Meanwhile, mortality rates between 10% and 14% have been published [19,20,21,23]. Despite the populations not being comparable, the rate of IMV was lower in this study, but the mortality rate was slightly higher compared to prior studies.

In the same way, few studies on the use of baricitinib for patients hospitalized with COVID-19 pneumonia have been published to date. In a retrospective study, no death was reported and only 1 patient was admitted to the ICU among 113 individuals treated with baricitinib [27]. However, included patients seemed to have moderate disease at the time of initiation of baricitinib based on oxygen saturation at presentation [27]. In a randomized trial, the incidence of progression to death or intubation in the first 28 days from admission was lower in the baricitinib plus remdesivir group vs. the remdesivir group (12.2% vs. 17.2%) [28]. However, almost 14% of included patients did not require supplemental oxygen (no death occurred on either arm in the baseline ordinal score 4 subgroup) [28]. In our study, baricitinib was combined with an antiviral in around 57% of patients. Less than 5% of the patients treated with baricitinib required invasive mechanical ventilation in our population, but the mortality rate was almost 17%, higher than that previously reported.

These differences in our survival results can be explained because individuals were censored at discharge from the hospital or on the date of death. So, data included complete hospitalization in conventional and intensive units’ periods. Several studies have censored patients with COVID-19 at the time of invasive mechanical ventilation or at 3 or 4 weeks after hospital admission. Therefore, their results cannot reflect the true mortality for COVID-19, because many of these patients may develop late complications and final outcomes were not collected. In fact, at day 21 after hospital admission, only 45% had been discharged from the hospital at the censored date in one of the anakinra studies [19]. However, in our study, 16 (69.6%) of the 23 individuals who needed invasive mechanical ventilation died after intubation. However, only 42 (13.2%) of the 319 subjects treated under immunomodulatory therapy in conventional hospitalization died. The overall ICU mortality rate of patients with COVID-19 in a systematic review was 30.6%, but when only mechanically ventilated subjects or acute respiratory distress syndrome subjects were considered, the mortality was 59% or up to 93%, respectively [33]. In our study, early interruption of anakinra or baricitinib occurred in most of the deceased individuals at the time of admission to the ICU. Therefore, we cannot rule out the possibility that the use of immunomodulatory drugs in critically ill patients would also be of benefit. In this setting, lower mortality was observed with the use of intravenous anakinra and concomitant corticosteroids in mechanically ventilated patients with COVID-19 in the ICU, but it was not statistically significant [22]. Moreover, in contrast to other studies, all included patients required supplemental oxygen support at admission. However, there were some differences among anakinra and baricitinib populations. Most patients in the anakinra group required a higher flow of oxygen support (category 5b or more) at the time of starting the immunomodulatory drug compared to the baricitinib group (70% vs. 34%, *p* < 0.001). These findings are in line with those of a recent clinical trial that showed anakinra to be inefficacious in mild COVID-19 patients [29]. By contrast, baricitinib, alone or in combination with antiviral drugs, could have early clinical benefits in the first days of infection or at the initial stages of the inflammatory phase [25]. Moreover, because venturi masks show a theoretically higher dispersion distance for aerosol particles [34], these delivery devices were not used in the anakinra group. However, during hospitalization, individuals treated with anakinra required less change in oxygen flow than those treated with baricitinib, with a difference of 9%. In both populations, we observed that the faster the drug is initiated in the management of patients with hypoxemic respiratory failure, the better the survival among patients with moderate or severe COVID-19. Prospective randomized studies will be necessary to dilucidated the best time to start this drug among hypoxemic patients with COVID-19.

Beneficial effects of corticosteroids have generally been found in patients with severe COVID-19 [4,5,6,35]. However, there is little information about the combination of corticosteroids with anakinra or baricitinib [22,26,28]. The clinical benefits of steroids might be related to the indication (severity of illness), timing of the intervention, and dose and duration of corticosteroid therapy [36]. In our study, all patients in both groups were treated with corticosteroids. Dexamethasone was extensively used in both groups at admission based on actual recommendations [4]. However, dexamethasone was changed to pulses of corticosteroids when worsening of clinical status occurred according to the physician’s criteria. In this sense, all patients were started on pulses of methylprednisolone, previous or concomitant to the initiation of anakinra. By contrast, high doses of corticosteroids were only used in 46% of subjects in the baricitinib group. We cannot rule out a deleterious effect when dexamethasone is dropped out and higher doses of corticosteroids are started. However, after propensity score matching, there were no significant differences between the use or no use of pulses of corticosteroids. A benefit of steroids, including high doses of corticosteroids, has been observed in the inflammatory phase of COVID-19 [4,5,6,35,36]. Moreover, we are concerned that the potential risk factor of higher doses of steroids in our global population could be associated to selection bias because they were used when clinical worsening was suspected.

Our study has several limitations. The most important is the retrospective design, with dynamic therapy recommendations over time. To reduce the bias due to confounding variables, propensity score matching was performed to adjust for baseline characteristics between cohorts. However, not many differences were found before and after matching. At the time of writing this paper, there was no significant evidence from clinical trials for the efficacy of anakinra or baricitinib in COVID-19 patients. The different strategies used in two close hospitals reflect the absence of global recommendations and the heterogeneous management during the pandemic. However, both therapies are based on drugs with short durations of action and effect, acceptable side-effect profiles, and ease of administration. Another important limitation is the lack of a concomitant control group. All the severe COVID-19 patients in the internal medicine ward were included for regimens based on immunomodulatory drugs. Only corticosteroids have been reported to have some clinical benefits [4,5,6], but this therapy was also used in all patients in both groups. Therefore, we cannot rule out a potential benefit of anakinra or baricitinib added to steroids based on the pathophysiology described in patients with COVID-19 [3]. Finally, complement system inhibition is also a potential therapeutic target for COVID-19 [8]. In this study, immunomodulatory drugs were used independently, but it will be interesting to investigate the potential role of the combination or sequential use of IL and JAK inhibitors in COVID-19.

In our experience, clinical, laboratory, and radiographic items should be considered when deciding on the use of immunomodulatory drugs in real life among patients with moderate or severe COVID-19. The exact time to start them may be related to their efficacy.

## 5. Conclusions

Similar mortality was observed in real life with a different strategy based on anakinra or baricitinib. Older age, low functional status, high comorbidity, a need for IMV, elevated LDH, and the use of a high flow of oxygen at admission were found to be related to the occurrence of major clinical events.

## Figures and Tables

**Table 1:** Modified WHO clinical progression scale. (5a) Supplemental oxygen (O_2_) by nasal cannula requiring low-flow oxygen (≤4 lpm); (5b) supplemental oxygen by nasal cannula requiring ≥5 lpm oxygen flow; (5c) supplemental oxygen by mask using FiO_2_ between 35% and 50%; (6a) supplemental oxygen by mask with a reservoir bag; (6b) supplemental oxygen by high-flow nasal cannula (HFNC); and (6c) non-invasive mechanical ventilation.

Patient State	Descriptor
5. Hospitalized moderate disease	(5a) Supplemental O_2_ by nasal cannula requiring ≤4 lpm flow
	(5b) Supplemental O_2_ by nasal cannula requiring ≥5 lpm flow
	(5c) Supplemental O_2_ by mask using FiO_2_ between 35% and 50%
6. Hospitalized severe disease	(6a) Supplemental O_2_ by mask with reservoir bag
	(6b) Supplemental O_2_ by high-flow nasal cannula (HFNC)
	(6c) Non-invasive mechanical ventilation

**Table 2:** Demographic, laboratory, and clinical data of patients in anakinra and baricitinib groups.

Variables	Anakinra Group (n = 125)	Baricitinib Group (n = 217)	*p* Univariate
Demographic data			
Age (median, IQR), in years	73 (59–78)	71 (59–82)	0.528
Male sex, *n* (%)	70 (56)	127 (58)	0.649
Charlson index > 2, *n* (%)	60 (48)	127 (58)	0.060
Barthel scale ≥ 60, *n* (%)	116 (93)	184 (85)	**0.030**
Cardiopulmonary resuscitation candidate, *n* (%)	97 (78)	157 (72)	0.285
Living in a nursing home, *n* (%)	3 (2)	11 (5)	0.230
Laboratory values (median, IQR)			
Ferritin, in ng/mL	746 (324–1329)	579 (299–1312)	0.577
D-dimers, in µg/mL	900 (550–1640)	1055 (595–2163)	0.936
C-reactive protein, in mg/L	103 (58–168)	98 (44–143)	**0.044**
Procalcitonin, in ng/mL	0.14 (0.08–0.23)	0.11 (0.07–0.20)	0.770
Erythrocyte sedimentation rate, in mm/h	42 (14–77)	51 (25–83)	0.057
Alanine aminotransferase, in U/L	30 (20–53)	26 (17–43)	**0.013**
Lactate dehydrogenase, in U/L	326 (254–414)	302 (230–387)	0.078
Platelets × 10^3^/µL	233 (174–305)	236 (157–320)	0.831
Lymphocytes/µL	880 (620–1265)	880 (620–1280)	0.390
Interleukin-6, in pg/mL *	16 (8–22)	20 (5–49)	**<0.001**
Time to event (median, IQR), in days			
Time of symptoms before admission	7 (5–10)	7 (5–10)	0.505
Time from admission to censored date	11 (8–15)	10 (7–16)	0.898
Time under first ID	10 (8–10)	8 (5–10)	0.239
Time from ID to combination event	7 (5–10)	9 (6–15)	**0.005**
Mean time from admission to ID	2.41	0.93	**<0.001**
Treatments, *n* (%)			
Remdesivir	2 (2)	35 (16)	**<0.001**
Lopinavir/ritonavir	0	77 (35)	**<0.001**
Dexamethasone at admission	77 (62)	172 (79)	**<0.001**
Pulses of corticosteroids at any time	125 (100)	99 (46)	**<0.001**
Changes in immunomodulatory therapy	5 (4)	31 (14)	**0.001**
Tocilizumab	1 (1)	13 (6)	**0.020**
Intermediate or high doses of LMWH ^1^	67 (57)	82 (38)	**<0.001**
Mask with reservoir bag at admission	61 (49)	22 (10)	**<0.001**

^1^ LMWH: low-molecular-weight heparin. *Available for 15 and 93 individuals.

**Table 3:** Multivariate Cox proportional analysis for the outcome of invasive mechanical ventilation.

Variables	Intubation	*p* Univariate	*p* Multivariate	Hazard Ratio (95% CI)
Lactate dehydrogenase				
≥350 U/L	15 (12.3)	0.002	0.015	2.907 (1.227–6.827)
<350 U/L	8 (3.6)			
Mask with reservoir bag				
Yes	13 (15.7)	<0.001	0.033	4.983 (1.141–21.770)
No	10 (3.9)			
Pulses of corticosteroids				
Yes	22 (9.9)	0.002	0.176	–
No	1 (0.8)			
C-reactive protein				
≥100	17 (10	0.017	0.241	–
<100	6 (3.5)			
Antiviral therapy				
Yes	4 (3.6)	0.110	0.228	–
No	19 (8.2)			
First immunomodulatory drug				
Anakinra	13 (10.4)	0.039	0.594	–
Baricitinib	10 (4.6)			
Dexamethasone at baseline				
Yes	8 (3.2)	<0.001	0.002	0.256 (0.108–0.611)
No	15 (16.1)			

**Table 4:** Multivariate Cox proportional analysis for the outcome of mortality.

Variables	Mortality	*p* Univariate	*p* Multivariate	Hazard Ratio (95% CI)
Lactate dehydrogenase				
≥350 U/L	26 (21.3)	0.110	0.199	–
<350 U/L	32 (14.5)			
Mask with reservoir bag				
Yes	25 (30.1)	<0.001	0.118	–
No	33 (12.7)			
Pulses of corticosteroids				
Yes	49 (22)	0.001	<0.001	1.668 (1.308–2.127)
No	9 (7.6)			
Age				
≥70	50 (25.6)	<0.001	<0.001	1.634 (1.279–2.087)
<70	8 (5.4)			
Dose of LMHW				
Prophylaxis	22 (12)	0.008	0.072	–
Intermediate or high dose	36 (22.8)			
Invasive ventilation				
Yes	16 (69.6)	<0.001	<0.001	12.576 (5.113–30.932)
No	42 (13.2)			
Barthel index				
<60	19 (45.2)	<0.001	0.037	1.604 (1.030–2.497)
≥60	39 (13)			
Charlson index				
<3	12 (7.7)	<0.001	0.076	–
≥3	46 (24.6)			

**Table 5:** Multivariate Cox proportional analysis for the outcome of mortality after propensity score matching.

Variables	Mortality	*p* Univariate	*p* Multivariate	Hazard Ratio (95% CI)
Lactate dehydrogenase				
≥350 U/L	18 (26.5)	0.062	0.188	–
<350 U/L	18 (15.3)			
Mask with reservoir bag				
Yes	18 (35.3)	0.001	0.003	2.949 (1.463–5.947)
No	18 (13.3)			
Pulses of corticosteroids				
Yes	31 (21.8)	0.125	0.289	–
No	5 (11.4)			
Age				
≥70	30 (27)	0.001	0.040	1.222 (1.024–3.932)
<70	6 (8)			
Dose of LMHW				
Prophylaxis	12 (13)	0.031	0.747	–
Intermediate or high dose	24 (25.5)			
Invasive ventilation				
Yes	10 (71.4)	<0.001	0.047	2.276 (1.011–6.360)
No	26 (15.1)			
Barthel index				
<60	12 (44.4)	<0.001	0.002	3.338 (1.559–7.150)
≥60	24 (15.1)			
Charlson index				
<3	5 (6.1)	<0.001	0.003	3.544 (1.330–9.441)
≥3	31 (29.8)			
First immunomodulatory drug				
Anakinra	15 (16.1)	0.265	0.631	–
Baricitinib	21 (22.6)			
Dexamethasone at baseline				
Yes	21 (16.7)	0.179	0.396	–
No	15 (25)			
C-reactive protein				
≥100	22 (23.7)	0.138	0.764	–
<100	14 (15.1)			

## Data Availability

The data presented in this study are available on request from the corresponding author.
